# Fistule urétro-rectale iatrogène

**DOI:** 10.11604/pamj.2020.37.161.20907

**Published:** 2020-10-15

**Authors:** Ahmed Ibrahimi, Idriss Ziani

**Affiliations:** 1 Service d´Urologie-A, Centre Hospitalo-Universitaire Ibn Sina, Faculté de Médecine et de Pharmacie, Université Mohammed V, 10000, Rabat, Maroc

**Keywords:** Fistule urétro-rectale, iatrogène, urétro-cystographie rétrograde, York-Mason, Urethro-rectal fistula, iatrogenic, retrograde urethrocystography, York-Mason

## Abstract

Urethro-rectal fistula is a rare pathological entity, most often iatrogenic secondary to prostate surgery, or traumatic secondary to trauma of the pelvis. We report the observation of a 73-year-old patient who consults in urology for urinary discharge through the anus during urination. He is a type I diabetic patient under poorly monitored insulin, he was treated for repeated urethritis, operated 4 months ago for Fournier gangrene with the notion of traumatic urethral catheterization, he presented after the removal of the bladder catheter a dysuria with emission of urine from the anus during urination which motivated a consultation. On clinical examination, the scrotum is supple and well healed after flattening the necrotic areas; absence of cutaneous fistulous pathways; in the digital rectal examination, a small induration is found at 6 cm from the anal margin class II according to the Rivera classification, with a firm prostate of 55 g. Retrograde urethrocystography (A, B, C, D) showed the presence of a urethro-rectal fistula with the presence of multiple moniliform narrowing of the anterior urethra in favor of chronic urethritis with muscular bladder and post-voiding residue. Trans-sphincter posterior surgical procedure according to the York-Mason technique has been proposed to treat this patient.

## Image en médecine

La fistule urétro-rectale (FUR) est une entité pathologique rare, le plus souvent iatrogène secondaire à la chirurgie de la prostate, ou traumatique secondaire aux traumatismes du bassin. Nous rapportons l´observation d´un patient âgé de 73 ans, consulté en urologie pour émission des urines par l´anus au moment de la miction. Il s´agit d´un patient diabétique de type I sous insuline mal suivi, il a été traité pour des urétrites à répétition, opéré il y a 4 mois pour gangrène de Fournier avec notion de sondage vésicale traumatique, il a présenté après l´ablation de la sonde vésicale une dysurie avec émission des urines par l´anus au moment de la miction ce qui a motivé une consultation. A l´examen clinique, le scrotum est souple bien cicatrisé après mise à plat des zones nécrosées; absence des trajets fistuleux cutanés; au toucher rectal on découvre une petite induration à 6 cm de la marge anale classé stade II selon la classification de Rivera, avec une prostate ferme de 55 g. L´urétro-cystographie rétrograde (UCG) (A, B, C, D) a montré la présence d´une fistule urétro-rectale avec présence de multiples rétrécissements moniliforme de l´urètre antérieur en faveur d´une urétrite chronique avec vessie de lutte et résidu post mictionnel. Une intervention chirurgicale par voie trans-sphinctérienne postérieure selon la technique de York-Mason a été proposée pour traiter ce patient.

**Figure 1:**
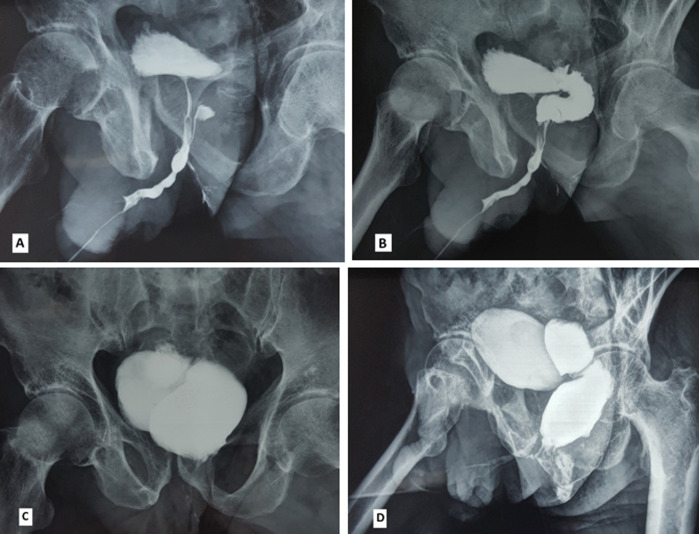
A) multiples rétrécissements moniliforme de l’urètre antérieur en faveur d’une urétrite chronique avec début d’opacification rectale et mise en évidence du trajet fistuleux; B) opacification rectal et mise en évidence d’une fistule urétro-rectal au temps de remplissage vésical; C) vessie pleine avec opacification de l’ampoule rectale; D) opacification du rectum et canal anal au temps mictionnel

